# Supplementary material for: Spatio-temporal regulation of endocytic protein assembly by SH3 domains in yeast
Source: Mol Biol Cell. 2023 Feb 21;34(3):ar19. doi: 10.1091/mbc.E22-09-0406 (PMC10011730; doi:10.1091/mbc.E22-09-0406)
Supplement: Supplementary file 5 [file mbc-34-ar19-s001.pdf]

Supplementary Materials

Molecular Biology of the Cell

Hummel and Kaksonen

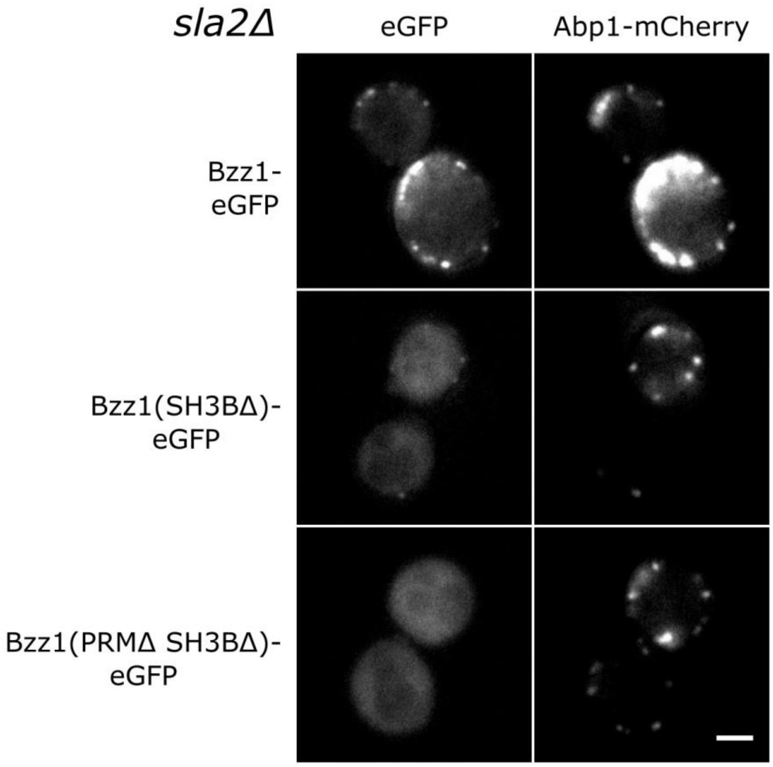

Figure S1: Bzz1 and Abp1 patch formation in *sla2Δ* cells. Images of representative cells are single frames of 3 min time-lapse movies and are representative examples of three independent experiments. Scale bar: 2 µm.

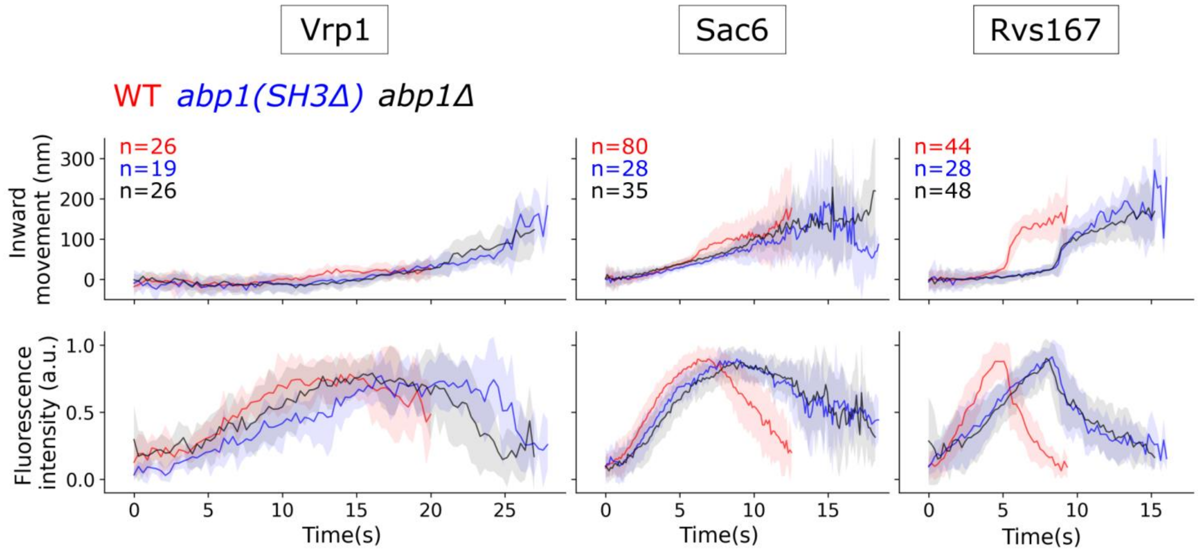

Figure S2: Deletion of *ABP1* causes the same phenotypes for Vrp1, Sac6 and Rvs167 as described for *abp1(SH3Δ)* cells in figure 3 and 4. Plots show median centroid movement and fluorescence intensities (solid lines) with median absolute deviations (shaded areas). All data depicted in this figure represent at least three independent experiments.

Table S1: Plasmid list

Name	Alias	Tag	Selection	Source
pMK0003	pFA6a-EGFP-HIS6MX	EGFP	hisMX6	Janke et al., 2004
pMK0005	pFA6a-mCherry-KanMX4	mCherry	kanMX4	Janke et al., 2004
pMK0019	pFA6a-natNT2	-	natNT2	Janke et al., 2004
pMK0052	pks133	-	hphNT1	Janke et al., 2004
pMK0086	pFA6a-KiURA	-	kiURA	Janke et al., 2004
pMK0099	pFA6a-EGFP-natNT2	EGFP	natNT2	gift from Michael Knop group
pMK0265	pFA6a-Bzz1-EGFP-HIS6MX	EGFP	hisMX6	this study
pMK0274	pFA6aBzz1(PAPEVPPPRR::SGGGG SGGGG)- EGFP-HIS6MX	EGFP	hisMX6	this study
pMK0275	pFA6a-Bzz1(W531A)-EGFP-HIS6MX	EGFP	hisMX6	this study
pMK0283	pFA6a-Bbc1-EGFP-HIS6MX	EGFP	hisMX6	this study
pMK0349	pFA6a-Bbc1(SH3Δ)-EGFP-HIS6MX	EGFP	hisMX6	this study

Janke C, Magiera MM, Rathfelder N, Taxis C, Reber S, Maekawa H, Moreno-Borchart A, Doenges G, Schwob E, Schiebel E, Knop M. 2004. **A versatile toolbox for PCR-based tagging of yeast genes: new fluorescent proteins, more markers and promoter substitution cassettes**. *Yeast* **21**:947–962. doi:10.1002/YEA.1142

Table S2: Strain list

Figure	Name	Genotype	Strain number
1	Bzz1-eGFP	MATa, his3-Δ200, leu2-3,112, ura3-52, lys2-801, BZZ1-EGFP::his3MX6	MKY2807
1	Bzz1(SH3AΔ PRMΔ SH3BΔ)-eGFP	MATa, his3-Δ200, leu2-3,112, ura3-52, lys2-801, BZZ1(1-492)-EGFP::his3MX6	MKY3639
1	Bzz1(SH3BΔ)-eGFP	MATa, his3-Δ200, leu2-3,112, ura3-52, lys2-801, BZZ1(1-576)-EGFP::his3MX6	MKY3699
1	Bzz1(PRMΔ SH3BΔ)-eGFP	MATa, his3-Δ200, leu2-3,112, ura3-52, lys2-801, BZZ1(1-560)-EGFP::his3MX6	MKY3636
1	Bzz1(W531A)-eGFP	MATa, his3-Δ200, leu2-3,112, ura3-52, lys2-801, BZZ1(W531A)-EGFP::his3MX6	MKY3972
1	Bzz1(PRM*)-eGFP	MATa, his3-Δ200, leu2-3,112, ura3-52, lys2-801, BZZ1(PAPEVPPPRR::SGGGGSGGGG)-EGFP::his3MX6	MKY3971

1	Bzz1-eGFP Abp1-mCherry	MATa, his3-Δ200, leu2-3,112, ura3-52, lys2-801, BZZ1-EGFP::his3MX6, ABP1- mCherry::kanMX4	MKY2806
1	Bzz1(SH3BΔ)-eGFP Abp1-mCherry	MATa, his3-Δ200, leu2-3,112, ura3-52, lys2-801, BZZ1(1-576)-EGFP::his3MX6, ABP1- mCherry::kanMX4	MKY3737
1	Bzz1(PRMΔ SH3BΔ)-eGFP Abp1-mCherry	MATa, his3-Δ200, leu2-3,112, ura3-52, lys2-801, BZZ1(1-560)-EGFP::his3MX6, ABP1- mCherry::kanMX4	MKY3729
1	Bzz1(W531A)-eGFP Abp1-mCherry	MATα, his3-Δ200, leu2-3,112, ura3-52, lys2-801, BZZ1(W531A)-EGFP::his3MX6, ABP1-mCherry::kanMX4	MKY3998
1	Bzz1(PRM*)-eGFP Abp1-mCherry	MATα, his3-Δ200, leu2-3,112, ura3-52, lys2-801, BZZ1(PAPEVPPPRR::SGGGGSGGGG)-EGFP::his3MX6, ABP1-mCherry::kanMX4	MKY3977
1	Bzz1-eGFP Abp1-mCherry *sla1Δ*	MATa, his3-Δ200, leu2-3,112, ura3-52, lys2-801, BZZ1-EGFP::his3MX6, ABP1- mCherry::kanMX4, sla1Δ::natNT2	MKY3766
1	Bzz1(SH3BΔ)-eGFP Abp1-mCherry *sla1Δ*	MATa, his3-Δ200, leu2-3,112, ura3-52, lys2-801, BZZ1(1-576)-EGFP::his3MX6, ABP1- mCherry::kanMX4, sla1Δ::natNT2	MKY3813
1	Bzz1(PRMΔ SH3BΔ)-eGFP Abp1-mCherry *sla1Δ*	MATa, his3-Δ200, leu2-3,112, ura3-52, lys2-801, BZZ1(1-560)-EGFP::his3MX6, ABP1- mCherry::kanMX4, sla1Δ::natNT2	MKY3762
2	Las17-eGFP Abp1-mCherry	MATa, his3-Δ200, leu2-3,112, ura3-52, lys2-801, LAS17-EGFP::his3MX6, ABP1- mCherry::kanMX4	MKY1367
2	Las17-eGFP Abp1-mCherry *sla1Δ*	MATa, his3-Δ200, leu2-3,112, ura3-52, lys2-801, LAS17-EGFP::his3MX6, ABP1- mCherry::kanMX4, sla1Δ::natNT2	MKY1875
2	Vrp1-eGFP Abp1-mCherry	MATa, his3-Δ200, leu2-3,112, ura3-52, lys2-801, VRP1-EGFP::his3MX6, ABP1- mCherry::kanMX4	MKY3900
2	Vrp1-eGFP Abp1-mCherry *sla1Δ*	MATα, his3-Δ200, leu2-3,112, ura3-52, lys2-801, VRP1-EGFP::his3MX6, ABP1- mCherry::kanMX4, sla1Δ::klURA3	MKY4002
2	Myo5-eGFP Abp1-mCherry	MATα, his3-Δ200, leu2-3,112, ura3-52, lys2-801, MYO5-EGFP::his3MX6, ABP1- mCherry::kanMX4	MKY0712
2	Myo5-eGFP Abp1-mCherry *sla1Δ*	MATα, his3-Δ200, leu2-3,112, ura3-52, lys2-801, MYO5-EGFP::his3MX6, ABP1- mCherry::kanMX4, sla1Δ::klURA3	MKY4073
2	Pan1-eGFP Abp1-mCherry	MATa, his3-Δ200, leu2-3,112, ura3-52, lys2-801, PAN1-EGFP::his3MX6, ABP1- mCherry::kanMX4	MKY1302
2	Pan1-eGFP Abp1-mCherry *sla1Δ*	MATa, his3-Δ200, leu2-3,112, ura3-52, lys2-801, PAN1-EGFP::his3MX6, ABP1- mCherry::kanMX4, sla1Δ::natNT2	MKY3822
2	Las17-eGFP Abp1-mCherry *sla1Δ bzz1Δ*	MATα, his3-Δ200, leu2-3,112, ura3-52, lys2-801, LAS17-EGFP::his3MX6, ABP1- mCherry::kanMX4, sla1Δ::klURA3, bzz1Δ::natNT2	MKY3922
2	Las17-eGFP Abp1-mCherry *sla1Δ syp1Δ*	MATα, his3-Δ200, leu2-3,112, ura3-52, lys2-801, LAS17-EGFP::his3MX6, ABP1- mCherry::kanMX4, sla1Δ::klURA3, syp1Δ::hphNT1	MKY1873
2	Las17-eGFP Abp1-mCherry *sla1Δ bbc1Δ*	MATa, his3-Δ200, leu2-3,112, ura3-52, lys2-801, LAS17-EGFP::his3MX6, ABP1- mCherry::kanMX4, sla1Δ::klURA3, bbc1Δ::cgLEU2	MKY3940
2	Las17-eGFP Abp1-mCherry *sla1Δ rvs167(SH3Δ)*	MATa, his3-Δ200, leu2-3,112, ura3-52, lys2-801, LAS17-EGFP::his3MX6, ABP1- mCherry::kanMX4, sla1Δ::klURA3, rvs167(SH3Δ)::natNT2	MKY3918

2	Las17-eGFP Abp1-mCherry *sla1Δ bzz1Δ rvs167(SH3Δ)*	MATa, his3-Δ200, leu2-3,112, ura3-52, lys2-801, LAS17-EGFP::his3MX6, ABP1- mCherry::kanMX4, sla1Δ::klURA3, bzz1Δ::natNT2, rvs167(SH3Δ)::natNT2	MKY3942
2	Las17-eGFP Abp1-mCherry *bzz1Δ*	MATα, his3-Δ200, leu2-3,112, ura3-52, lys2-801, LAS17-EGFP::his3MX6, ABP1- mCherry::kanMX4, bzz1Δ::natNT2	MKY4032
2	Las17-eGFP Abp1-mCherry *rvs167(SH3Δ)*	MATa, his3-Δ200, leu2-3,112, ura3-52, lys2-801, LAS17-EGFP::his3MX6, ABP1- mCherry::kanMX4, rvs167(SH3Δ)::natNT2	MKY3919
2	Las17-eGFP Abp1-mCherry *bzz1Δ rvs167(SH3Δ)*	MATa, his3-Δ200, leu2-3,112, ura3-52, lys2-801, LAS17-EGFP::his3MX6, ABP1- mCherry::kanMX4, bzz1Δ::natNT2, rvs167(SH3Δ)::natNT2	MKY3970
3A	Abp1-eGFP	MATα, his3-Δ200, leu2-3,112, ura3-52, lys2-801, ABP1-EGFP::his3MX6	MKY2834
3B	Abp1-eGFP	MATα, his3-Δ200, leu2-3,112, ura3-52, lys2-801, ABP1-EGFP::natNT2	MKY4035
3	Abp1(SH3Δ)-eGFP	MATa, his3-Δ200, leu2-3,112, ura3-52, lys2-801, ABP1(1-531)-EGFP::his3MX6	MKY3997
3	Sac6-eGFP	MATa, his3-Δ200, leu2-3,112, ura3-52, lys2-801, SAC6-EGFP::his3MX6	MKY3864
3	Sac6-eGFP *abp1(SH3Δ)*	MATa, his3-Δ200, leu2-3,112, ura3-52, lys2-801, SAC6-EGFP::his3MX6, abp1(1- 531)::natNT2	MKY4127
3	Cap1-eGFP	MATa, his3-Δ200, leu2-3,112, ura3-52, lys2-801, CAP1-EGFP::his3MX6	MKY2865
3	Cap1-eGFP *abp1(SH3Δ)*	MATa, his3-Δ200, leu2-3,112, ura3-52, lys2-801, CAP1-EGFP::his3MX6, abp1(1- 531)::natNT2	MKY4140
3	Sla1-eGFP Sac6-mCherry	MATa, his3-Δ200, leu2-3,112, ura3-52, lys2-801, SLA1-EGFP::his3MX6, SAC6- mCherry::kanMX4	MKY4015
3	Sla1-eGFP Sac6-mCherry *abp1(SH3Δ)*	MATa, his3-Δ200, leu2-3,112, ura3-52, lys2-801, SLA1-EGFP::his3MX6, abp1(1- 531)::natNT2, SAC6-mCherry::kanMX4	MKY4214
3	Pan1-eGFP Sac6-mCherry	MATa, his3-Δ200, leu2-3,112, ura3-52, lys2-801, PAN1-EGFP::his3MX6, SAC6- mCherry::kanMX4	MKY4017
3	Pan1-eGFP Sac6-mCherry *abp1(SH3Δ)*	MATa, his3-Δ200, leu2-3,112, ura3-52, lys2-801, PAN1-EGFP::his3MX6, abp1(1- 531)::natNT2, SAC6-mCherry::kanMX4	MKY4223
3	Ark1-eGFP	MATa, his3-Δ200, leu2-3,112, ura3-52, lys2-801, ARK1-EGFP::his3MX6	MKY4181
3	Ark1-eGFP *abp1(SH3Δ)*	MATa, his3-Δ200, leu2-3,112, ura3-52, lys2-801, ARK1-EGFP::his3MX6, abp1(1- 531)::natNT2	MKY4200
3	Prk1-eGFP	MATα, his3-Δ200, leu2-3,112, ura3-52, lys2-801, PRK1-EGFP::his3MX6	MKY4131
3	Prk1-eGFP *abp1(SH3Δ)*	MATa, his3-Δ200, leu2-3,112, ura3-52, lys2-801, PRK1-EGFP::his3MX6, abp1(1- 531)::natNT2	MKY4158
3	Inp52-eGFP	MATa, his3-Δ200, leu2-3,112, ura3-52, lys2-801, INP52-EGFP::his3MX6, ABP1- mCherry::kanMX4	MKY3623

3	Inp52-eGFP *abp1(SH3Δ)*	MATα, his3-Δ200, leu2-3,112, ura3-52, lys2-801, INP52-EGFP::his3MX6, abp1(1- 531)::natNT2	MKY4218
4	Rvs167-eGFP	MATα, his3-Δ200, leu2-3,112, ura3-52, lys2-801, RVS167-EGFP::his3MX6	MKY2832
4	Rvs167-eGFP *abp1(SH3Δ)*	MATα, his3-Δ200, leu2-3,112, ura3-52, lys2-801, RVS167-EGFP::his3MX6, abp1(1- 531)::natNT2	MKY4213
4	Aim21-eGFP	MATα, his3-Δ200, leu2-3,112, ura3-52, lys2-801, AIM21-EGFP::his3MX6	MKY4133
4	Aim21-eGFP *abp1(SH3Δ)*	MATα, his3-Δ200, leu2-3,112, ura3-52, lys2-801, AIM21-EGFP::his3MX6, abp1(1- 531)::natNT2	MKY4162
4	Myo5-eGFP	MATα, his3-Δ200, leu2-3,112, ura3-52, lys2-801, MYO5-EGFP::his3MX6	MKY3870
4	Myo5-eGFP *abp1(SH3Δ)*	MATα, his3-Δ200, leu2-3,112, ura3-52, lys2-801, MYO5-EGFP::his3MX6, abp1(1- 531)::natNT2	MKY4219
4	Vrp1-eGFP	MATa, his3-Δ200, leu2-3,112, ura3-52, lys2-801, VRP1-EGFP::his3MX6	MKY3860
4	Vrp1-eGFP *abp1(SH3Δ)*	MATa, his3-Δ200, leu2-3,112, ura3-52, lys2-801, VRP1-EGFP::his3MX6, abp1(1- 531)::natNT2	MKY4212
4	Las17-eGFP Sac6-mCherry	MATa, his3-Δ200, leu2-3,112, ura3-52, lys2-801, LAS17-EGFP::his3MX6, SAC6- mCherry::kanMX4	MKY4021
4	Las17-eGFP Sac6-mCherry *abp1(SH3Δ)*	MATa, his3-Δ200, leu2-3,112, ura3-52, lys2-801, LAS17-EGFP::his3MX6, abp1(1- 531)::natNT2, SAC6-mCherry::kanMX4	MKY4225
5	Lsb3-eGFP Abp1-mCherry	MATa, his3-Δ200, leu2-3,112, ura3-52, lys2-801, LSB3-EGFP:: his3MX6, ABP1- mCherry::kanMX4	MKY4451
5	Lsb3(SH3Δ)-eGFP Abp1-mCherry	MATa, his3-Δ200, leu2-3,112, ura3-52, lys2-801, LSB3(SH3Δ)-EGFP:: his3MX6, ABP1- mCherry::kanMX4	MKY4475
5	Bbc1-eGFP Abp1-mCherry	MATα, his3-Δ200, leu2-3,112, ura3-52, lys2-801, BBC1-EGFP:: his3MX6, ABP1- mCherry::kanMX4	MKY4425
5	Bbc1(SH3Δ)-eGFP Abp1-mCherry	MATα, his3-Δ200, leu2-3,112, ura3-52, lys2-801, BBC1(SH3Δ)-EGFP:: his3MX6, ABP1-mCherry::kanMX4	MKY4307
S1	Bzz1-eGFP Abp1-mCherry *sla2Δ*	MATa, his3-Δ200, leu2-3,112, ura3-52, lys2-801, BZZ1-EGFP::his3MX6, ABP1- mCherry::kanMX4, sla2Δ::natNT2	MKY3889
S1	Bzz1(SH3BΔ)-eGFP Abp1-mCherry *sla2Δ*	MATa, his3-Δ200, leu2-3,112, ura3-52, lys2-801, BZZ1(1-576)-EGFP::his3MX6, ABP1- mCherry::kanMX4, sla2Δ::natNT2	MKY3897
S1	Bzz1(PRMΔ SH3BΔ)-eGFP Abp1-mCherry *sla2Δ*	MATa, his3-Δ200, leu2-3,112, ura3-52, lys2-801, BZZ1(1-560)-EGFP::his3MX6, ABP1- mCherry::kanMX4, sla2Δ::natNT2	MKY3887
S2	Vrp1-eGFP *abp1Δ*	MATa, his3-Δ200, leu2-3,112, ura3-52, lys2-801, VRP1-EGFP::his3MX6, abp1Δ::klURA3	MKY4232
S2	Sac6-eGFP *abp1Δ*	MATα, his3-Δ200, leu2-3,112, ura3-52, lys2-801, SAC6-EGFP::his3MX6, abp1Δ::klURA3	MKY4126
S2	Rvs167-eGFP *abp1Δ*	MATa, his3-Δ200, leu2-3,112, ura3-52, lys2-801, RVS167-EGFP::his3MX6, abp1Δ::klURA3	MKY4230
