# Supplementary material for: Spatio-temporal regulation of endocytic protein assembly by SH3 domains in yeast
Source: Mol Biol Cell. 2023 Feb 21;34(3):ar19. doi: 10.1091/mbc.E22-09-0406 (PMC10011730; doi:10.1091/mbc.E22-09-0406)
Supplement: Supplementary file 6 [file mbc-34-ar19-s002.pdf]

Spatio-temporal regulation of endocytic protein assembly by SH3 domains in yeast

Daniel Hummel and Marko Kaksonen

Corresponding author(s): Marko Kaksonen, University of Geneva

Review Timeline:

Submission Date:	2022-09-07
Editorial Decision:	2022-11-02
Revision Received:	2022-12-23
Accepted:	2022-12-24

Editor-in-Chief: Matthew Welch

Transaction Report:

(Note: With the exception of the correction of typographical or spelling errors that could be a source of ambiguity, letters and reports are not edited. The original formatting of letters and referee reports may not be reflected in this compilation.)

RE: Manuscript #E22-09-0406
TITLE: Spatio-temporal regulation of endocytic protein assembly by SH3 domains in yeast

Dear Marko,

Your manuscript has been reviewed by two experts in the field. You will see that both find your study interesting. However, they have some important suggestions for strengthening your manuscript. I think you should be able to address them without too much difficulty.

Sincerely,
Laurent

Laurent Blanchoin
Monitoring Editor
Molecular Biology of the Cell

Dear Dr. Kaksonen,

The review of your manuscript, referenced above, is now complete. The Monitoring Editor has decided that your manuscript is not acceptable for publication at this time, but may be deemed acceptable after specific revisions are made, as described in the Monitoring Editor's decision letter above and the reviewer comments below.

A reminder: Please do not contact the Monitoring Editor directly regarding your manuscript. If you have any questions regarding the review process or the decision, please contact the MBoC Editorial Office (mboc@ascb.org).

When submitting your revision include a rebuttal letter that details, point-by-point, how the Monitoring Editor's and reviewers' comments have been addressed. (The file type for this letter must be "rebuttal letter"; do not include your response to the Monitoring Editor and reviewers in a "cover letter.") Please bear in mind that your rebuttal letter will be published with your paper if it is accepted, unless you haveopted out of publishing the review history.

Authors are allowed 90 days to submit a revision. If this time period is inadequate, please contact us at mboc@ascb.org.

Revised manuscripts are assigned to the original Monitoring Editor whenever possible. However, special circumstances may preclude this. Also, revised manuscripts are often sent out for re-review, usually to the original reviewers when possible. The Monitoring Editor may solicit additional reviews if it is deemed necessary to render a completely informed decision.

In preparing your revised manuscript, please follow the instruction in the Information for Authors (www.molbiolcell.org/info-for-authors). In particular, to prepare for the possible acceptance of your revised manuscript, submit final, publication-quality figures with your revision as described.

To submit the rebuttal letter, revised manuscript, and figures, use this link: Link Not Available

Please contact us with any questions at mboc@ascb.org.

Thank you for submitting your manuscript to Molecular Biology of the Cell. We look forward to receiving your revised paper.

Sincerely,

Eric Baker
Journal Production Manager
MBoC Editorial Office
mbc@ascb.org

Reviewer #1 (Remarks to the Author):

The recruitment and regulation of many endocytic factors are mediated by interactions between SH3 and poly-P domains. The involvement of multiple proteins makes it difficult to understand the contribution of each SH3 domain in this complex interaction network. This work completes our knowledge in the field by focusing on the SH3 domains of proteins less studied until now. The general interest of this work is to show that these SH3 domains do not present a universal function. They can participate either in the recruitment and accumulation of the protein of which they are part of, or in the recruitment and anchoring of additional factors. They can also participate in the activation of these factors or in their inhibition, sometimes by competition for the same binding site. This study provides new and interesting information for specialists in this field.

I'm wondering about the Bzz1(W531A) mutant which is the only one behaving more or less like the WT protein. Is it possible that this mutation is not exactly comparable to a deletion of the SH3 domain? Is it possible to perform a similar mutation for SH3B and SH3B PRM to verify that the phenotypes are similar?
It's also surprising that SH3A and SH3B are so different as the Tonikian/Boone data predict that the two SH3 domains of Bzz1 should display interactions with similar poly-proline sequences, which seems contradictory with the results from this study. Is it possible for the authors to verify whether the observed difference comes from the position of the SH3 domains rather than any structural difference? For example, could the authors switch SH3A and SH3B to test this idea? Otherwise could the authors determine how/why SH3A interacts specifically with the actin network?

I also regret that the fluorescence intensity data are systematically normalized in this study (as in other studies from the same laboratory). I feel that a lot of important information is lost with such a representation of the data. For example, it is difficult to assess what fraction of WT or mutant proteins is actually recruited to the membrane (whether at individual patches or in total to the membrane) or left in the cytoplasm. Also, when such data are normalized, how is it possible to compare the rate of recruitment of each factor to the membrane (for example WT versus SH3B)? This representation of the data indicates a recruitment rate relative to the total amount of protein recruited, which may be very different from the accumulation rate of that protein.

Reviewer #2 (Remarks to the Author):

The authors should be commended for taking on such a challenging issue: how complex interactions among SH3 domain-containing proteins and their targets influence each other's recruitment, spatial organization, and function within a large macromolecular complex. They systematically dissect this problem at endocytic sites in budding yeast using mutants and live imaging, and uncover new relationships and effects, which they summarize nicely in their model. Overall, the experiments are straight forward, the data are clean, and the story provides an important advance to the field. Below are revisions I believe will strengthen the paper to ready it for publication.

Minor points:
1. Line 151. Cite original paper showing Sla1 and Bzz1 compete for binding to Las17.
2. Line 178. The paragraph ends with a colon, when it should be a period.
3. Bottom of page 6, explain what 'cytoplasmic' Las17 patches are, and we know they are cytoplasmic. Not evident from looking at the images. Also, how dynamic are these cytoplasmic Las17 patches?
4. Line 196. Reference original work showing Syp1 and Bbc1 regulate Las17 (Rodal et al., 2003; Boettner et al., 2009).
5. Line 198. Same issue, please cite original work showing Rvs167 phenotype.
6. Line 237 and 239. It will be confusing to non-expert readers to use "Las17/Vrp1" or "Bzz1/Rvs167". This suggests they are two names for the same protein. I suggest "Las17+Vrp1" and "Bzz1+Rvs167", or something else.

Major points:
7. In each figure legend, the authors should state how many times they independently performed experiments leading to the data shown.
8. Figure1C. Bar graph should have error bars.
9. Figure 2. The authors should demonstrate how they reach the conclusion that Abp1 forms "cytoplasmic clusters". Also, use arrows in the images to distinguish cortical patches from cytoplasmic clusters.
10. Line 286-289. Statements are made about longer patch lifetimes, but no quantification is shown. What are the average lifetimes and error bars?
11. One of the most interesting findings of the paper is that abp1ΔSH3 mutants have many important phenotypes, never seen before. Nice job! However, this raises a new question which I think should be addressed: how many of these abp1ΔSH3 phenotypes are observed in abp1Δ? If abp1Δ, phenotypes are less severe than those of abp1ΔSH3, it might indicate dominant or gain of function effects for abp1ΔSH3. The paper would not lose any meaning or importance, but this would be an important clarifier before publication, otherwise it will linger in the field for years as an unanswered question. Perhaps the authors could just pick a few of the key changes they see in abp1ΔSH3 mutants, and repeat those for abp1Δ.
12. Discussion. In light of their new findings on Abp1, it would be helpful to the field if the authors could speculate about why

abp1Δ is synthetic lethal with sla1Δ, sla2Δ, and sac6Δ. Also, it might be worth mentioning that abp1ΔSH3 is synthetic lethal with sla1Δ, sla2Δ, and sac6Δ (Quintero et al., 2005).
13. Discussion. Could the authors comment on how/why actin nucleation ends at endocytic sites, allowing LVC-associated proteins to dissociate?
14. Discussion. In the final paragraph, the authors make an assumption, "As SH3 domains typically have only one binding site..." to then make an argument. However, I would caution them against making this assumption, as SH3 domains also can have many non-canonical interactions, thus allowing them to bind more than one ligand at a time via a combination of canonical and non-canonical binding surfaces (reviewed in many places, e.g., Kaneko et al., 2008; PMID 18508559; and Saksela and Permi, 2012; PMID 22710157).

We thank both reviewers for their positive and constructive feedback, which has helped to improve our manuscript. Our detailed replies to all the reviewers' comments are given below in blue font.

Reviewer #1 (Remarks to the Author):

The recruitment and regulation of many endocytic factors are mediated by interactions between SH3 and poly-P domains. The involvement of multiple proteins makes it difficult to understand the contribution of each SH3 domain in this complex interaction network. This work completes our knowledge in the field by focusing on the SH3 domains of proteins less studied until now. The general interest of this work is to show that these SH3 domains do not present a universal function. They can participate either in the recruitment and accumulation of the protein of which they are part of, or in the recruitment and anchoring of additional factors. They can also participate in the activation of these factors or in their inhibition, sometimes by competition for the same binding site. This study provides new and interesting information for specialists in this field.

I'm wondering about the Bzz1(W531A) mutant which is the only one behaving more or less like the WT protein.

The apparent similarity between the Bzz1(W531A) mutant with Bzz1 (WT) was a result of the normalization of the fluorescence intensities. We have therefore quantified the relative intensities of the Bzz1 mutants, as suggested in this reviewer’s comment below. We measured the peak patch intensities and re-scaled the TIRF data according to these peak intensities in fig. 1B. Now, one can see that on average the Bzz1(W531A) intensity is indeed similar to WT, but that the intensity of the mutant varies much more than WT. The mutation seems to cause heterogeneity in the total amount of Bzz1 recruited to endocytic sites.

Is it possible that this mutation is not exactly comparable to a deletion of the SH3 domain? Is it possible to perform a similar mutation for SH3B and SH3B PRM to verify that the phenotypes are similar?

The Bzz1 SH3B is at the Bzz1 terminus. Thus, truncating the SH3B entirely was the simplest way of testing if the SH3B regulates Bzz1 assembly. In case of an internal domain, like Bzz1 SH3A, we chose a point mutation to keep the relative position of the other Bzz1 domains intact. It is true that the SH3A point mutant might have remaining non-canonical interactions, unlike the full deletion of the SH3B. However, we did not aim to describe the endocytic SH3 domains on the level of exact SH3 binding surfaces. While non-canonical interactions are an interesting aspect of the endocytic SH3 domains, we think it is out of the scope of this work.

It's also surprising that SH3A and SH3B are so different as the Tonikian/Boone data predict that the two SH3 domains of Bzz1 should display interactions with similar poly-proline sequences, which seems contradictory with the results from this study. Is it possible for the authors to verify whether the observed difference comes from the position of the SH3 domains rather than any structural difference? For example, could the authors switch SH3A and SH3B to test this idea?

Dionne et al. showed that the position of an SH3 domain indeed influences this SH3 domain's interactions, using Sla1 as an example protein. In addition to positional specificity of SH3 domains, however, Dionne et al. could also show that the Bzz1 SH3A and SH3B have intrinsically different specificities. Replacement of these SH3 domains with "stuffer" sequences lead to interactions that suggest that the Bzz1 SH3A and SH3B have some common interactions but also unique ones. When the Bzz1 SH3 domains were put into another protein, each had some unique interaction partners. To conclude the findings from Dionne et al., it seems the combination of position and sequence of an SH3 domain both defines an SH3 specificity. Tonikian/Boone had a different experimental approach and studied the Bzz1 SH3 domains as single domains and their preferential binding to short peptides. Hence, Tonikian/Boone describe the preferential binding motif

of the canonical binding site of the Bzz1 SH3 domains, while Dionne et al. cover also non-canonical interactions but do not describe a preferential binding motif. We agree that the question about the similarity of Bzz1 SH3A and SH3B should be addressed, but we think this deserves a Bzz1-specific study following up our findings.

Otherwise could the authors determine how/why SH3A interacts specifically with the actin network?

The endocytic actin phase is a multi-factorial event concerning protein-protein interactions. There are rearrangements of protein positions, assembly of new proteins and formation of membrane curvature. The Latrunculin A treatment inhibits all three aspects. To dissect, if the Bzz1 SH3A binds to a protein that assembles in an actin-dependent fashion or if another aspect of the actin phase enables Bzz1 SH3A binding, we tested patch formation of Bzz1, Bzz1(SH3BΔ) and Bzz1(PRMΔ SH3BΔ) in *sla2Δ* cells (fig. S1). In *sla2Δ* cells, tethering of the actin network to the membrane is perturbed so that the plasma membrane remains flat while the actin network grows (Kaksonen et al., 2003). Thus, if the Bzz1 SH3A regulates Bzz1 patch formation in *sla2Δ* cells, positional rearrangements and membrane curvature could be excluded as Bzz1 SH3A interaction-promoting factors, and consequently we could test different binding candidates like actin-binding proteins by gene deletions. However, as shown in fig. S1, Bzz1 and Bzz1(SH3BΔ) patches localize to the plasma membrane, but Bzz1(PRMΔ SH3BΔ) does not. This suggests that the SH3A interaction requires invagination growth and thus is dependent on functional actin phase and not simply on the presence of the proteins of the actin network as protein-protein interaction partners. The answer to the question how/why SH3A interacts specifically with the actin network seems to be complex. The combination of the results in our LatA and *sla2Δ* experiments implies that the Bzz1 SH3A binding partner/site becomes available during invagination growth. Perhaps, the competition between Sla1 and the Bzz1 SH3A changes when invagination growth occurs, so that the SH3A can bind. In addition, binding of the Bzz1 F-BAR to curved membrane could regulate the Bzz1 SH3A via intramolecular interactions.

I also regret that the fluorescence intensity data are systematically normalized in this study (as in other studies from the same laboratory). I feel that a lot of important information is lost with such a representation of the data. For example, it is difficult to assess what fraction of WT or mutant proteins is actually recruited to the membrane (whether at individual patches or in total to the membrane) or left in the cytoplasm. Also, when such data are normalized, how is it possible to compare the rate of recruitment of each factor to the membrane (for example WT versus SH3B)? This representation of the data indicates a recruitment rate relative to the total amount of protein recruited, which may be very different from the accumulation rate of that protein.

We agree with this concern and now present the data scaled to true intensity values. This kind of scaling is difficult to do directly for TIRF data, as TIRF imaging is very sensitive to illumination angle, cell wall thickness, etc. Therefore, we measured peak intensities of Bzz1 mutant patches by epifluorescence imaging to scale the fluorescence intensities from the TIRF microscopy experiments (fig. 1B). The data shows that the Bzz1 SH3B regulates Bzz1 assembly before the actin phase and that the SH3B strongly promotes Bzz1 peak molecule numbers at endocytic sites. The Bzz1 PRM also regulates Bzz1 assembly before the actin phase but does not seem to affect the Bzz1 peak molecule numbers much. On average, the Bzz1 SH3A mutant looks similar to Bzz1 WT, but the mutant shows a strong variability concerning peak intensities. We thank the reviewer for this suggestion to strengthen our manuscript.

Reviewer #2 (Remarks to the Author):

The authors should be commended for taking on such a challenging issue: how complex interactions among SH3 domain-containing proteins and their targets influence each other's recruitment, spatial organization, and function within a large macromolecular complex. They systematically dissect this problem at endocytic sites in budding yeast using mutants and live imaging, and uncover new relationships and effects, which they summarize nicely in their model. Overall, the experiments are straight forward, the data are clean, and the story provides an important advance to the field. Below are revisions I believe will strengthen the paper to ready it for publication.

Minor points:
1. Line 151. Cite original paper showing Sla1 and Bzz1 compete for binding to Las17.
2. Line 178. The paragraph ends with a colon, when it should be a period.

We thank the reviewer for pointing out these corrections; we have made all of these changes.

3. Bottom of page 6, explain what 'cytoplasmic' Las17 patches are, and we know they are cytoplasmic. Not evident from looking at the images. Also, how dynamic are these cytoplasmic Las17 patches?

We imaged the cells in the equatorial plane and found three types of patches. Persistent patches at the plasma membrane, transient patches at the plasma membrane and transient patches that are not associated to the plasma membrane. The latter type of patches was called "transient patches in the cytoplasm" when we described this phenotype for the first time in the text. Later in the text, we just called them ‘cytoplasmic’. To clarify this description, we have changed the text to make the description of these different types of patches clearer and the naming consistent. In the figure 2A, we added green arrows pointing to the "transient patches in the cytoplasm" for Las17-eGFP and Myo5-eGFP. As these images were taken from the equatorial plane of the cells, the highlighted patches are distinguishable from patches at the plasma membrane.

The cytoplasmic patches appear dynamic, but in this experiment, the acquisition rate was kept low so that we could take long videos without too much photo bleaching. Hence, we do not know if these patches turn over or just move in and out of the focal plane. They are abnormal structures that are not present in WT cells and are gone in *sla1Δ bzz1Δ*; therefore, we did not characterize their dynamic behavior in detail.

4. Line 196. Reference original work showing Syp1 and Bbc1 regulate Las17 (Rodal et al., 2003; Boettner et al., 2009).
5. Line 198. Same issue, please cite original work showing Rvs167 phenotype.
6. Line 237 and 239. It will be confusing to non-expert readers to use "Las17/Vrp1" or "Bzz1/Rvs167". This suggests they are two names for the same protein. I suggest "Las17+Vrp1" and "Bzz1+Rvs167", or something else.

We thank the reviewer for pointing out these corrections. We now cite the original papers and use “Las17-Vrp1”.

Major points:7. In each figure legend, the authors should state how many times they independently performed experiments leading to the data shown.

We added this information to each figure legend.

8. Figure1C. Bar graph should have error bars.

This is now corrected. The bar graph now represents mean and standard deviation of three independent experiments.

9. Figure 2. The authors should demonstrate how they reach the conclusion that Abp1 forms "cytoplasmic clusters". Also, use arrows in the images to distinguish cortical patches from cytoplasmic clusters.

We added arrows in the figure and a supplemental video (Figure 3 – video 1).

10. Line 286-289. Statements are made about longer patch lifetimes, but no quantification is shown. What are the average lifetimes and error bars?

We now describe WT lifetimes more quantitatively so that the difference to the mutant phenotype is clearer (see bottom of page 10).

11. One of the most interesting findings of the paper is that abp1ΔSH3 mutants have many important phenotypes, never seen before. Nice job! However, this raises a new question which I think should be addressed: how many of these abp1ΔSH3 phenotypes are observed in abp1Δ? If abp1Δ, phenotypes are less severe than those of abp1ΔSH3, it might indicate dominant or gain of function effects for abp1ΔSH3. The paper would not lose any meaning or importance, but this would be an important clarifier before publication, otherwise it will linger in the field for years as an unanswered question. Perhaps the authors could just pick a few of the key changes they see in abp1ΔSH3 mutants, and repeat those for abp1Δ.

We agree with this point and we analyzed patch dynamics of Vrp1, Sac6 and Rvs167 in *abp1Δ* cells. The phenotypes for these proteins look identical in both *abp1(SH3Δ)* and *abp1Δ* (see fig. S2). Hence, there does not seem to be dominant or gain of function effects for *abp1(SH3Δ)*, but instead Abp1's main functions appear to be dependent on the Abp1 SH3 domain.

12. Discussion. In light of their new findings on Abp1, it would be helpful to the field if the authors could speculate about why abp1Δ is synthetic lethal with sla1Δ, sla2Δ, and sac6Δ. Also, it might be worth mentioning that abp1ΔSH3 is synthetic lethal with sla1Δ, sla2Δ, and sac6Δ (Quintero et al., 2005).

We now address this topic in a separate section of the Discussion (line 471 – 489).

13. Discussion. Could the authors comment on how/why actin nucleation ends at endocytic sites, allowing LVC-associated proteins to dissociate?

We added a paragraph addressing this question to the Discussion. We speculate about potential mechanisms in the context of our SH3 interaction network model (line 457 – 469).

14. Discussion. In the final paragraph, the authors make an assumption, "As SH3 domains typically have only one binding site..." to then make an argument. However, I would caution them against making this assumption, as SH3 domains also can have many non-canonical interactions, thus allowing them to bind more than one ligand at a time via a combination of canonical and non-canonical binding surfaces (reviewed in many places, e.g., Kaneko et al., 2008; PMID 18508559; and Saksela and Permi, 2012; PMID 22710157).

We agree that our wording was too simplistic and we have corrected the paragraph.

RE: Manuscript #E22-09-0406R
TITLE: "Spatio-temporal regulation of endocytic protein assembly by SH3 domains in yeast"

Dear Marko:

I am pleased to accept your manuscript for publication in Molecular Biology of the Cell.

Sincerely,
Laurent Blanchoin
Monitoring Editor
Molecular Biology of the Cell

Dear Dr. Kaksonen:

Congratulations on the acceptance of your manuscript.

A PDF of your manuscript will be published on MBoC in Press, an early release version of the journal, within 10 days. The date your manuscript appears at www.molbiolcell.org/toc/mboc/0/0 is the official publication date. Your manuscript will also be scheduled for publication in the next available issue of MBoC.

Within approximately four weeks you will receive a PDF page proof of your article.

Would you like to see an image related to your accepted manuscript on the cover of MBoC? Please contact the MBoC Editorial Office at mboc@ascb.org to learn how to submit an image.

Authors of Articles and Brief Communications are encouraged to create a short video abstract to accompany their article when it is published. These video abstracts, known as Science Sketches, are up to 2 minutes long and will be published on YouTube and then embedded in the article abstract. Science Sketch Editors on the MBoC Editorial Board will provide guidance as you prepare your video. Information about how to prepare and submit a video abstract is available at www.molbiolcell.org/science-sketches. Please contact mboc@ascb.org if you are interested in creating a Science Sketch.

We are pleased that you chose to publish your work in MBoC.

Sincerely,

Eric Baker
Journal Production Manager
MBoC Editorial Office
mbc@ascb.org
